# AR Signaling in Human Malignancies: Prostate Cancer and Beyond

**DOI:** 10.3390/cancers10010022

**Published:** 2018-01-18

**Authors:** Emmanuel S. Antonarakis

**Affiliations:** The Sidney Kimmel Comprehensive Cancer Center at Johns Hopkins, 1650 Orleans Street, CRB1–1M45, Baltimore, MD 21287, USA; eantona1@jhmi.edu; Tel.: +1-443-287-0553

## Abstract

The notion that androgens and androgen receptor (AR) signaling are the hallmarks of prostate cancer oncogenesis and disease progression is generally well accepted. What is more poorly understood is the role of AR signaling in other human malignancies. This special issue of *Cancers* initially reviews the role of AR in advanced prostate cancer, and then explores the potential importance of AR signaling in other epithelial malignancies. The first few articles focus on the use of novel AR-targeting therapies in castration-resistant prostate cancer and the mechanisms of resistance to novel antiandrogens, and they also outline the interaction between AR and other cellular pathways, including PI3 kinase signaling, transcriptional regulation, angiogenesis, stromal factors, Wnt signaling, and epigenetic regulation in prostate cancer. The next several articles review the possible role of androgens and AR signaling in breast cancer, bladder cancer, salivary gland cancer, and hepatocellular carcinoma, as well as the potential treatment implications of using antiandrogen therapies in these non-prostatic malignancies.

Androgens and androgen receptor (AR) signaling are the hallmarks of prostate cancer oncogenesis and disease progression. While the medical literature is saturated by studies examining the role of androgens/AR in prostate cancer, less attention has been given to the potential importance of the AR pathway in other human malignancies. The goal of this special issue of *Cancers* is to shed more light on the clinical significance of androgen/AR signaling, not just in prostate cancer, but also in other epithelial malignancies.

This theme issue begins with a thoughtful summary by Schweizer et al. [1] introducing the AR signaling field in prostatic and other malignancies. After describing the biological and therapeutic roles of AR in prostate cancer, the authors review the evidence supporting AR-directed therapies in other tumor types including breast cancer, bladder cancer, kidney cancer, pancreatic cancer, hepatocellular cancer, ovarian and endometrial cancers, mantle cell lymphoma, and salivary gland cancers. This is followed by a review by Crumbaker et al. [2] that summarizes the interaction between AR and PI3 kinase signaling in prostate cancer, outlines the role of the PI3K pathway in prostate cancer, and reviews the potential clinical utility of dual targeting of AR and PI3K as a therapeutic strategy in prostate cancer. The next review by Obinata et al. [3] delves deeper into the interplay between AR and other collaborative transcription factors (such as FOXA1, GATA2, and OCT1), and proposes new strategies to co-target AR together with some of these transcriptional collaborators, with particular attention to pyrrole–imidazole polyamide as a candidate compound. This is followed by a review article by Eisermann et al. [4] discussing the interactions between AR, angiogenesis, and the vascular endothelial growth factor (VEGF) in prostate cancer, hormone-mediated mechanisms of VEGF regulation, and potential therapeutic strategies that take into account both AR and hypoxia as potential regulators of angiogenesis. The next article, by Leach et al. [5], reviews the important but understudied subject of AR signaling in the stromal compartment (primarily in fibroblasts and myofibroblasts) in the context of prostate cancer, suggesting that stromal AR activity strongly influences prognosis and progression of this disease. The next article, by Cucchiara et al. [6], summarizes our knowledge of epigenomic regulation of AR in prostate cancer, discusses the various types of epigenetic control (including DNA methylation, chromatin modification, and noncoding RNAs), and ends with some therapeutic implications including the use of the demethylase inhibitor SD-70. Finally, the article by Pakula et al. [7] reviews our current understanding of the interaction between AR and Wnt pathway signaling in prostate cancer, the central role of beta-catenin in this context, and possible therapeutic applications of drugs that target both AR and Wnt/beta-catenin pathways in prostate cancer.

The second series of articles begins to address the role of AR signaling in other human cancers, with a focus on potential therapeutic implications. Rahim et al. [8] begin with a thoughtful overview of the role of androgens and AR signaling in breast cancer (especially in triple-negative breast cancer), they summarize the biology and prognostic/predictive role of AR in breast cancer, and they end with some thoughts on potential therapeutic strategies. This is followed by a second review article on this topic by Narayanan et al. [9] who delve deeper into the therapeutic strategies (nonsteroidal agonists and antagonists) that target androgen/AR signaling in breast cancer. Asano et al. [10] then present an original research article investigating protein expression (by immunohistochemistry) of the AR molecule in 190 cases of triple-negative breast cancer, showing that positive AR protein expression in triple-negative breast cancer tissues is associated with a better prognosis and should perhaps be used to sub-classify cases of triple-negative disease for prognostic purposes. Next, Li et al. [11] review the current knowledge of AR signaling in urothelial carcinoma of the bladder, summarize the data linking androgens to urothelial carcinogenesis and tumor growth, and offer some chemopreventive and therapeutic options for bladder cancer management. After this, the article by Dalin et al. [12] reviews the data on AR signaling in salivary gland cancer (particularly salivary duct carcinoma), and summarizes the prevalence, biology, and therapeutic implications of AR signaling in salivary gland cancers. Finally, the last article in this special issue, by Kanda et al. [13], reviews the role of AR in hepatocellular cancer, its centrality in the development of this malignancy, the potential role of AR in regulating the innate immune response in this disease, and strategies combining sorafenib with AR inhibitors for therapeutic purposes.

We hope that the readership enjoys this this special issue of *Cancers*, that they become informed about the role of androgens and AR signaling in the context of multiple different cancer types, and that this treatise will ignite further clinical research and therapeutic trials aiming to modulate the AR pathway in various human malignancies.
